# Behavioral and Neurophysiological Correlates of Dogs’ Individual Sensitivities to Being Observed by Their Owners While Performing a Repetitive Fetching Task

**DOI:** 10.3389/fpsyg.2020.01461

**Published:** 2020-07-15

**Authors:** Orsolya Kiss, Anna Kis, Katalin Scheiling, József Topál

**Affiliations:** ^1^Institute of Cognitive Neuroscience and Psychology, Research Centre for Natural Sciences, Budapest, Hungary; ^2^Department of Cognitive Science, Budapest University of Technology and Economics, Budapest, Hungary; ^3^Faculty of Psychology and Neuroscience, Maastricht University, Maastricht, Netherlands

**Keywords:** dog (*Canis familiaris*), dog–human interaction, audience effect, relationship insecurity, sleep EEG

## Abstract

Ample evidence suggests that dogs possess enhanced skills in reading human visual attention, but it remains to be explored whether they are sensitive to the audience effect in their interactions with humans. The present study aimed to investigate how dogs’ behavior is affected by their owners’ visual attention while performing a repetitive task (bringing an object back to an unfamiliar experimenter while the owner waited passively). We assumed that if dogs are susceptible to the audience effect, their task persistence and task performance would vary according to their owners’ attentiveness. A group of adult pet dogs (*N* = 27) were repeatedly presented with an object retrieval task by the experimenter (*N* = 20 trials) while owners either ignored their dogs (Inattentive Owner condition) or paid attention to their dogs’ actions (Attentive Owner condition). Behavioral observations were complemented with the owner’s reports of their relationships with their dogs (assessed by means of an owner–pet attachment questionnaire) and dogs’ spectral EEG sleep profile (recorded during 3-h-long daytime sleep). Although dogs, independently of their owners’ attentional state, were generally willing to comply with the fetching task, they were faster to approach the toy object and gazed significantly longer at their owners when he/she was paying attention. This finding is reminiscent of peer influence observed in humans. Further, characteristics of relationship insecurity (relationship anxiety and avoidance) were associated with dogs’ task persistence and performance. Dogs of owners with higher relationship anxiety tended to approach the toy object less frequently, and dogs of owners with higher relationship avoidance and anxiety were more hesitant to approach the toy object. We also found that dogs’ individual susceptibilities to the audience effect is related to EEG spectral power of both REM and non-REM sleep as well as in pre-sleep (drowsiness) in a trait-like manner. These results, in line with previous findings, support the notion that dogs have a somewhat human-like susceptibility to the audience effect, a trait which might be linked to more complex mechanisms, such as self-presentation or reputation management, helping the two species to become effective social partners.

## Introduction

The ability to monitor the focus of others’ visual attention has crucial importance in human social functioning. Since the early experiments of Triplett (1898) a large body of research has been initiated in order to understand and explain the impact of social presence on human behavior. The general finding is that when people think they are being watched, they are less likely to break the social rules (Baillon et al., 2013) and more open for cooperation (Burnham and Hare, 2007). But the phenomenon of the “audience effect” is not restricted to humans; it can also be used to describe social interaction between non-human animals (Coppinger et al., 2017). Ample evidence suggests that the mere presence of conspecifics may affect the behavior of non-human animals in various situations. Social influences can both inhibit and facilitate behavior among group mates as has been reported in wide range of animals, including non-human primates (Visalberghi and Addessi, 2000; Reynaud et al., 2015), other mammals (Sherman, 1977), birds (Evans and Marler, 1994), and fish (Karplus et al., 2006).

Primates, however, respond not just to the mere presence of others, but also to their visual attention. Sensitivity to the visual attention of others is important because it allows group mates to gain information about each other’s activities and potential cooperation. Increasing evidence suggests that non-human primates are able to adjust their behavior to others’ attention state, and thus, they have at least a basic understanding of “being watched,” an important precondition for the emergence of human-like features of the audience effect. For example, olive baboons (*Papio anubis*) adjust their requesting gestures to the state of the eyes (open/closed) of a potential helper (Bourjade et al., 2015). It has also been shown that orangutans (*Pongo pygmaeus*) modify their facial expressions when a recipient is watching them (Waller et al., 2015), and gibbons (*Hylobates* sp.) use their facial expressions differentially depending on the attentional state of others (Scheider et al., 2016).

Although communication usually involves interactions between conspecifics, domestic dogs (*Canis familiaris*) represent a special case among animals as they are not only adept at communicating with conspecifics, but can engage in communication with people (Kaminski and Marshall-Pescini, 2014). Dogs are able to use social signals effectively and purposefully in dog–human interactions, including expressive use of vocalization (Miklósi et al., 2000; Horowitz and Bekoff, 2007), body posture (Quaranta et al., 2007), and visual attention cues (Virányi et al., 2006). Moreover, dogs readily follow human gestural signals (Miklósi and Soproni, 2006) from 6 weeks of age onward (Riedel et al., 2006) and can extract information from vocal intonation cues (Colbert-White et al., 2018). Even though little is known about the socio-cognitive abilities of domesticated species other than dogs, it has been reported that horses (*Equus caballus*) (Maros et al., 2008), domestic cats (*Felis catus*) (Miklósi et al., 2005), goats (*Capra hircus*) (Kaminski et al., 2005), and pigs (*Sus scrofa domestica*) (Nawroth et al., 2014, 2016) are also able to follow certain types of human pointing. These findings highlight the role of domestication as a special evolutionary process that might have caused substantial changes in attention allocation and willingness to cooperate with humans. Dogs also seem to possess those two basic skills that are necessary to respond adequately when being watched: They are sensitive to changes in their partner’s visual attention and are able to use the emotional information provided by a human partner. It has been reported, for example, that dogs can take into account the visual access of their human partner in a fetching task (visual perspective-taking; Kaminski et al., 2009) and are less likely to engage in forbidden behavior when the human is looking at them (Schwab and Huber, 2006; Kaminski et al., 2013). Dogs can distinguish between attentive and inattentive human partners and not only recognize human facial expressions (e.g., Siniscalchi et al., 2018), but they also use facial changes in response to changing attention of their human audience (Kaminski et al., 2017). There is also some evidence that they tend to use their owners’ affective cues to guide their own behavior toward novel objects (Merola et al., 2012, 2014; Turcsán et al., 2015) in problem-solving tasks. Evidence also suggests that dogs’ human-directed behavior (i.e., gazing at the human, approaching a human) is affected not only by social familiarity (Horn et al., 2013), but by the social aspects of the dog–human relationship and the owner’s interaction styles toward his/her dog (Topál et al., 1997; Horn et al., 2012). Namely, the specific relationship that a dog has with its human audience influences its attention toward that person.

Most research revolving around the audience effect focuses on the group-level phenomena (i.e., members of certain species react in a certain way to being observed under different conditions). However, as in case of all other socio-cognitive capacities, individual variability can be observed regarding sensitivity to the presence of others. It is, thus, plausible to assume that such variability is related to neurophysiological parameters. Sleep EEG fingerprints are one promising such parameter that have been shown in humans to correlate with individual variability in several domains. For example, attachment patterns (Sloan et al., 2007) and IQ (Ujma et al., 2014, 2015) have been robustly shown to be related to sleep EEG parameters. This means that, while no cause–effect conclusions can be drawn from these correlative studies, there is a significant covariation between the behavioral and the neural measures at the individual level. There are also examples that are potentially relevant to the audience effect phenomena. In addition to tracking the audience engagement (attention level), judging others’ emotional reactions is also crucial to one who is observed. Evidence suggests that problems in recognizing and interpreting other people’s emotional expressions can lead to poor interpersonal functioning (Shimokawa et al., 2001). Recent studies have revealed an interesting feature of human emotion recognition ability: Emotion recognition and responsiveness to social-affective signals are particularly sensitive to sleep quality (for a review, see Beattie et al., 2014). For example, sleep duration was found to be associated with peer acceptance and social engagement, two components of peer social competence (Vaughn et al., 2015). Sleep disturbance can lead to impaired social interactions (Gilbert et al., 2015) and reduce self-expression in social interactions (Condén et al., 2013) and has the potential to reduce the accuracy of identifying facial expressions of happiness and sadness (Crönlein et al., 2016; Killgore et al., 2017). The effect of sleep deprivation on facial emotion identification was also confirmed by the results of resting state EEG studies. Findings suggest associations among poor emotional processing, left lateralization of alpha power, and increased ratio of the power density (theta/beta ratio) in the frontal area (Zhang et al., 2019). It has also been reported that atypical features of REM sleep physiology (reduced REM sleep gamma EEG activity) predict decreased emotional reactivity (van der Helm et al., 2011).

Personality traits and attachment style are additional important factors in social responsiveness (West and Sheldon-Kellor, 1994), and individual differences in personality factors and attachment have well-documented associations with several sleep parameters. For example, objective measures of sleep quality (e.g., alpha intrusion in non-REM sleep, a marker of hyperarousal during sleep) are associated with attachment anxiety but not with attachment avoidance (Sloan et al., 2007). Studies also showed significant associations between sleep quality and the “big five” personality traits: There is a negative relationship among subjective measures of sleep quality and neuroticism (emotional lability; Calkins et al., 2013), conscientiousness (Williams and Moroz, 2009), and extraversion (Blagrove and Akehurst, 2001).

There is only scarce and circumstantial evidence for the associations between dogs’ responsiveness to social-affective signals and sleep parameters. Non-invasive polysomnography studies on dogs showed significant differences in the spectral characteristics of sleep EEG between the active and passive day (Kis et al., 2014). Dogs also display considerable individual variation in sleep macrostructure as measured by sleep efficiency, sleep latency, sleep cycle duration, slow wave sleep, and REM sleep time (Kis et al., 2014), and there are age- and sex-related differences in sigma burst activity during non-REM sleep (Iotchev et al., 2019). However, although the effects of pre-sleep emotions on dogs’ subsequent sleep have also been reported in dogs (Kis et al., 2017), very little (if any) is known about the associations between the individual differences in emotion processing, social responsiveness, and sleep.

The current study, therefore, investigated the effects of a human audience on dogs’ performance during a repetitive fetching task. More specifically, we aimed to examine the impact of the owner’s visual attention on dogs’ tendency to bring back an object to an unfamiliar experimenter and to investigate the potential associations among the owner–dog relationship, dogs’ task performance and spectral EEG sleep profile. We predicted that a dog’s willingness to perform a repetitive fetching task would change in response to the changing attentional state of its owner who is passively watching his/her dog. Namely, we would expect dogs to perform better in a repetitive task when they are being watched than when being ignored by their owners. We would also expect associations between the different aspects of the owners’ relationships with their dogs (pet-related anxiety and avoidance) and dogs’ sensitivity to their owners’ visual attention. Lower scores for pet-related anxiety and avoidance may be associated with better task performance. We also aimed to unravel potential associations between dogs’ sleep EEG spectrum and their susceptibility to the audience effect, but due to the exploratory nature of this investigation, we refrained from putting forward any specific hypothesis.

## Materials and Methods

### Ethics Statement

This research was conducted in accordance with the Hungarian regulations on animal experimentation and the guidelines for the use of animals in research described by the Association for the Study Animal Behavior (ASAB). Ethical approvals were obtained from the National Animal Experimentation Ethics Committee for both non-invasive EEG recordings (Ref No. PEI/001/1057-6-2015) and behavioral observations (Ref No. PE/EA/853-2/2016). Owners of the pet dogs participated in the study on a voluntary basis and gave their consent for EEG recordings as well as the behavioral testing of their dogs.

### Subjects

Twenty-seven adult pet dogs (18 females and 9 males; mean age: 4.46 years, SD: 2.21) and their owners participated in the test. Dogs were from 22 different breeds and 4 mongrels. Since the experiment was built on the task for dogs to bring back a toy object, only subjects that had been trained to retrieve objects on command were studied.

### Experimental Procedures

#### Behavioral Testing

The experiment took place in a room (5 m × 6 m) at the Institute of Cognitive Neuroscience and Psychology. One chair for the owner and some toys for the dog were placed in the room. The tests were video-recorded from four different angles (using cameras fixed to the walls). Before the trials began, the dogs were led into the room by their owners and allowed to explore the room for 5 min.

The experimental procedure consisted of two phases: (1) *Toy preference test* and (2) *Fetching task.* Phase (1) merely served to choose the toy that motivated the dog, while in phase (2), dogs’ behavior was examined in a repetitive fetching task situation, comparing two conditions: when the owner showed attention (Attentive Owner condition) vs. when the owner did not watch (Inattentive Owner condition).

##### Toy preference test

The experimenter briefly explained the tasks and asked which command the dog was familiar with for bringing back the toy. Then the experimenter familiarized herself with the dog: walked with it and initialized a fetching/rolling game with the dog: called its name and presented three different types of toys. Based on the dog’s preference, one toy—the one the dog picked to play with the most—was selected for the experiment, and the rest of the toys were removed from the room.

##### Fetching task

The owner held the dog on a leash at the starting point, and the experimenter verbally attracted the dogs’ attention to the toy object (“Look, here!”) while holding the toy visibly in her hand. Then she placed the toy at a predetermined point on the floor (3 m from the starting point) and went back to the starting point, took the leash from the owner, and asked her/him to take a seat.

**FIGURE 1:**
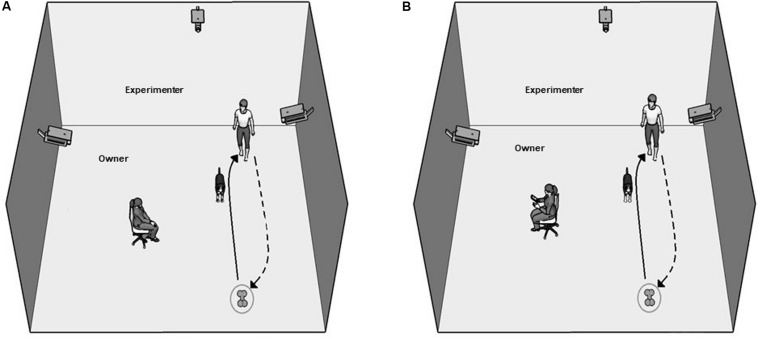
Experimental arrangement in the Attentive **(A)** and Inattentive **(B)** Owner conditions. The path of the experimenter, when she places the toy, is indicated with the arrows.

Attentive Owner (AO) condition (Figure 1A): In this condition, the chair faced the field where the dog and the experimenter were. The owner was asked to remain passively in his/her sitting position and to watch the dog silently but attentively. At the moment when the owner sat down and took up his/her position, the experimenter instructed the dog to fetch the toy using a command that the dog was familiar with. The command was repeated once every 5 s until the dog fetched the toy but no more than five times. The dog was praised by the experimenter when it brought back the toy, and the trial was terminated. If, however, the dog did not bring back the toy even after the fifth command, the trial was also terminated. Note, that if the dog refrained from approaching the toy (within 0.5 m) even after the fifth repeated command, the trial was labeled as “Refused.”

Inattentive Owner (IO) condition (Figure 1B): In this condition, dogs participated in the same procedure as in AO except that the owner’s chair was turned around, making the owner face the wall. Furthermore, the owner was instructed to read (a book or mobile phone) and ignore the dog throughout the trial.

The order of IO and AO trials was predetermined and semirandomized so that there were no more than two consecutive trials of the same type. Dogs received a maximum of 20 trials in a single session (10 IO and 10 AO trials in total; *N* = 21 dogs). However, if a dog performed three consecutive “Refused” trials in both IO and AI each, the *Fetching task* was finished (*N* = 3 dogs completed only 9, 12, and 13 trials). Moreover, three additional dog–owner pairs gave up further participation in the *Fetching task* before reaching the criterion of 2 × 3 consecutive “Refused” trials; these dogs completed 6, 7, and 14 trials. The whole procedure was video-recorded and analyzed later by two independent observers.

#### Questionnaire Data Collection

Before the behavioral observations, owners were asked to fill in a questionnaire assessing dog–owner relationship. This 16-item questionnaire was originally developed by Beck and Madresh (2008), and each item was rated on a Likert scale (1–7). The questionnaire includes 16 questions to assess two scales of human–dog relationship insecurity: 8 items for relationship anxiety (Pet-related Anxiety Scale – PANXS) and 8 items for relationship avoidance (Pet-related Avoidance Scale – PAVS). Generally speaking, PANXS relates to the owner’s worries about the quality and the future of his/her relationship with the dog, and PAVS relates to expectations about the dog as trustworthy and supportive. These two different aspects of the owners’ relationships with their dogs were calculated by summarizing the scores of the variables representing each trait. Cronbach’s alpha was used to assess the internal consistency of the factors (α = 0.646 for PANXS and α = 0.644 for PAVS).

#### Sleep EEG Recordings

Dogs also participated in 3-h-long daytime sleep measurements following the protocol described in Kis et al. (2014). Sleep EEG recordings were performed in a sleep laboratory (2 m × 3 m) either prior to the behavioral observations (on the same day: *N* = 13 dogs, 10–220 days before behavioral observations: *N* = 9 dogs) or 2–30 days later (*N* = 5 dogs). The timing of the recording could vary depending on the preferences of the participating dog owners but was restricted to the period between 12 pm and 6 pm as dogs show the highest propensity to sleep during the afternoon (apart from nighttime; Takahashi et al., 1972). The sleep laboratory was equipped with a mattress on the floor, and owners could decide whether they preferred their dog to sleep on the mattress with them or on the floor next to them. There were no windows in the room in order to ensure constant light conditions, but a table lamp was provided for the owners to read during the measurement. Dogs were allowed a 5–10 min exploration and familiarization and then the owner took place on the mattress and assisted the experimenter throughout the process of fixing surface attached electrodes onto the dog. The dog was rewarded with food during electrode placement if the owner deemed it necessary, social reinforcement (praise, petting) was used in all cases.

The following electrodes were used: Fz and Cz on the anteroposterior midline of the skull as well as F7 and F8 placed bilaterally on the zygomatic arch. A common reference was used for all four electrodes at the Pz position (posterior end of the skull midline). The ground electrode (G) was placed on the left musculus temporalis. Signals were prefiltered, amplified, and digitized at a sampling rate of 1,024 Hz/channel by using the SAM 25?R style MicroMed Headbox (MicroMed Inc, Houston, TX, United States) with hardware passband at 0.5–256 Hz, sampling rate of 512 Hz, anti-aliasing filter with cutoff frequency at 1 kHz, and 12-bit resolution covering a voltage range of ±2 mV as well as second-order software filters at 0.016 Hz (high pass) and 70 Hz (low pass) using System Plus Evolution software (MicroMed Inc., Houston, TX, United States). In addition, electrocardiogram (ECG), respiration, and muscle tone was monitored in order to aid sleep stage identification. Impedances for the EEG electrodes were kept below 20 kΩ.

### Behavior Variables

Behavioral data were analyzed by frame-by-frame coding of all experimental recordings (with a 0.2-s resolution, using Solomon Coder (beta 091110, ©2006 by András Péter^1^). The following behavior variables (16) were recorded:

(1) Latency to approach the toy, LAT_Appr/Toy_: The time (s) elapsed between the moment when the experimenter instructed the dog to fetch the toy and the moment when the dog arrived at the toy (its paw/muzzle was closer than 50 cm to the toy).
(2) Latency to give the toy over to the experimenter, LAT_Give/Toy__/Exp_: The time (s) elapsed between the moment when the experimenter instructed the dog to fetch the toy and the moment when the experimenter took the toy in her hand.
(3) Latency to give the toy over to the owner, LAT_Give/Toy/Own_: The time (s) elapsed between the moment when the experimenter instructed the dog to fetch the toy and the moment when the dog approached (<0.5 m) the owner with the toy in his mouth.
(4) Gazing at the owner, WATCH_*Own*_: Relative duration (t%) of the head orientation toward the owner.
(5) Gazing at the experimenter, WATCH_Exp_: Relative duration (t%) of the head orientation toward the experimenter.
(6) Latency of first gaze at the owner, LAT_WatchOwn_: The time (s) elapsed between the moment the experimenter instructed the dog to fetch the toy and the moment of the dog’s first head orientation toward the owner.
(7) Latency of first gaze at the experimenter, LAT_WatchExp_: The time (s) elapsed between the moment when the experimenter instructed the dog to fetch the toy and the moment of the dog’s first head orientation toward the experimenter.
(8) Time spent close to the experimenter, PROX_Exp_: The percentage of the total time (t%) spent in close proximity (<0.5 m) to the experimenter.
(9) Time spent close to the owner, PROX_*Own*_: The percentage of the total time (t%) spent in close proximity (<0.5 m) to the owner.
(10) Time spent close to the toy, PROX_Toy_: The percentage of the total time (t%) spent in close proximity (<0.5 m) to the toy object.
(11) Whether the dog approached the toy during the trial (Yes/No), Binary_Appr_.
(12) The total number of trials during which the dog approached (<50 cm) the toy, N_Appr_.
(13) Whether the dog brought back the toy during the trial (Yes/No), Binary_*Fetch*__/__Exp_.
(14) The total number of trials during which the dog brought back the toy to the experimenter, N_*Fetch*__/__Exp_.
(15) Whether the dog tried to involve the owner during the trial (Yes/No), Binary_*Fetch/Own*_.
(16) The total number of trials during which the dog tried to involve the owner in the task (i.e., tried to give the toy to the owner), N_*Fetch*__/__*Own*_.

Two additional variables were used to analyze questionnaire data (Pet-related Avoidance- and Anxiety Scales); see above.

Sleep EEG recordings were visually scored in accordance with standard criteria in 20-s epochs (see Kis et al., 2014, for a more detailed description) identifying the following stages: wakefulness, drowsiness, non-REM, and REM sleep. Artifact rejection was carried out by visual inspection on 4-s epochs using the EEG viewing program Fercio’s EEG Plus (©Ferenc Gombos 2009–2017) before further automatic analyses. Average power spectral densities (1–30 Hz) were calculated by a mixed-radix fast Fourier transformation (FFT) algorithm, applied to the 50% overlapping, Hanning-tapered 4-s windows of the EEG signal for the Fz, Cz, F7, and F8 derivations respectively. Relative spectral power values for the different vigilance states (drowsiness, non-REM, and REM) were calculated for each for each frequency bin with 0.25 Hz resolution by dividing the absolute power of the given frequency bin with the total spectral power (on the full 1–30 Hz spectrum).

### Statistical Analysis

First we used Wilcoxon matched-pairs signed rank tests to analyze dogs’ willingness to participate in the fetching task: (i) the number of trials in which they approached the toy object and (ii) the number of trials in which they brought it back were compared between Attentive and Inattentive Owner conditions.

Then Pearson’s correlation analysis was performed to evaluate the strength of association between some of the abovementioned behavior variables (latency measures, durations of gazing, and whereabouts of dogs; see variables 1–10 above). After having removed the uninformative (redundant) variables from further analyses, dogs’ “Fetching Task” behavior was analyzed with random intercept generalized linear mixed-effect models (GLMM, IBM SPSS 23). The models included a random grouping factor (subject IDs), two fixed factors (Condition – Attentive vs. Inattentive Owner; Numerical order of trials – from 1 up to maximum 20) and covariates (Pet-related Avoidance and Anxiety scales) as well as all combinations of two-way interactions. Non-significant effects were removed from the model in a stepwise manner (backward elimination technique). Statistical tests were two-tailed, and α value was set at 0.05.

In order to assess the relationship between dogs’ sensitivity to being watched and sleep parameters, we calculated difference scores between Attentive and Inattentive Owner conditions for all behavioral variables (selected after eliminating redundant variables). These difference scores were then correlated with partial correlations with the relative spectrum using a bin-by-bin analysis on the full (1–30 Hz) spectrum with 0.25 Hz resolution, factoring in the time between sleep and behavioral measurements. In order to address the issue of multiple comparisons, we used the procedure of descriptive data analysis delineating the so-called Rüger’s areas (Abt, 1987). Rüger’s areas are defined as sets of conventionally significant (*p* < 0.05) results, which are accepted or rejected as significant as a whole instead of individual results of statistical tests. Taking the results of the statistical tests as a matrix, we defined Rüger’s areas along the dimension of frequency bins. Starting from the lower frequencies, a Rüger’s area is the range of all the neighboring, consecutive frequency bins that contain a significant result surrounded by bins containing non-significant results. After defining these areas of significance, the number of significant results within the area was calculated, and it was investigated whether at least half of these results were significant at least at 1/2 of the conventional *p* = 0.05 significance level (that is, whether they were below 0.025) and at least one third of them were significant at least at 1/3 of the conventional *p* = 0.05 significance level (that is, whether they were below 0.0167). If both of these conditions were fulfilled, the area as a whole was considered significant. With this method, a single significant statistical test with *p* < 0.0167 theoretically counts as a significant Rüger’s area; however, we would not have considered single-bin results as an area.

## Results

### Dogs’ Tendency to Participate in Fetching Task

Dogs performed similarly in the Attentive and Inattentive Owner conditions in terms of the number of trials in which they approached the toy object [Wilcoxon signed-rank test, Z_(__9__)_ = −0.77, *p* > 0.05; Median/AO = 10, IQR/AO = 2; Median/IO = 10, IQR/IO = 3]. The majority of subjects approached the toy in every trial (*N* = 17 and 18 dogs in AO and IO conditions, respectively), and each one of the 27 dogs approached the toy at least once in both conditions. Dogs also performed comparably in the two conditions in terms of the number of trials in which they brought back the toy to the experimenter [Wilcoxon signed-rank test, Z_(__14__)_ = -1.35, *p* > 0.05; Median/AO = 7, IQR/AO = 9; Median/IO = 6, IQR/IO = 9], the majority of subjects retrieved the toy at least once in both conditions (*N* = 23 in AO and *N* = 21 in IO).

### Reducing the Number of Redundant Behavioral Variables

There were significant correlations between the variables related to the latency to approach the toy and give it over to the experimenter and/or to the owner (LAT_Appr/Toy_ – LAT_Give/Toy/Exp_, Pearson’s *r* = 0.352; LAT_Appr/Toy_ – LAT_Give/Toy/Own_, *r* = 0.765; LAT_Give/Toy/Own_ – LAT_Give/Toy/Exp_, *r* = 0.392, *p* < 0.01 for all). Therefore, only one of these (*Latency to approach the toy*) was included in the GLMM analysis. Moreover, since the relative duration of gazing also significantly correlated with dogs’ latency of first gaze at the owner and at the experimenter respectively (WATCH_*Own*_ – LAT_WatchOwn_, *r* = -0.142; WATCH_Exp_ – LAT_WatchExp_, *r* = -0.358, *p* < 0.01 for both), only the relative duration of gazing at the owner and at the experimenter (WATCH_*Own*_ and WATCH_Exp_) were retained for further analysis. Time spent close to the experimenter, owner, and toy also were not included in the GLMM analyses because these variables significantly correlated with each other (PROX_Exp_ – PROX_*Own*_, *r* = -0.270; PROX_*Own*_ – PROX_Toy_, *r* = -0.374; PROX_Exp_ – PROX_Toy_ *r* = -0.151; *p* < 0.01 for all), and these variables also significantly correlated with the relative duration of gazing at the owner (WATCH_*Own*_). The results of the correlation analyses are summarized in Supplementary Table S1.

### The Effects of Owners’ Attention and Questionnaire Scales on Dogs’ Fetching Task Performance

**FIGURE 2:**
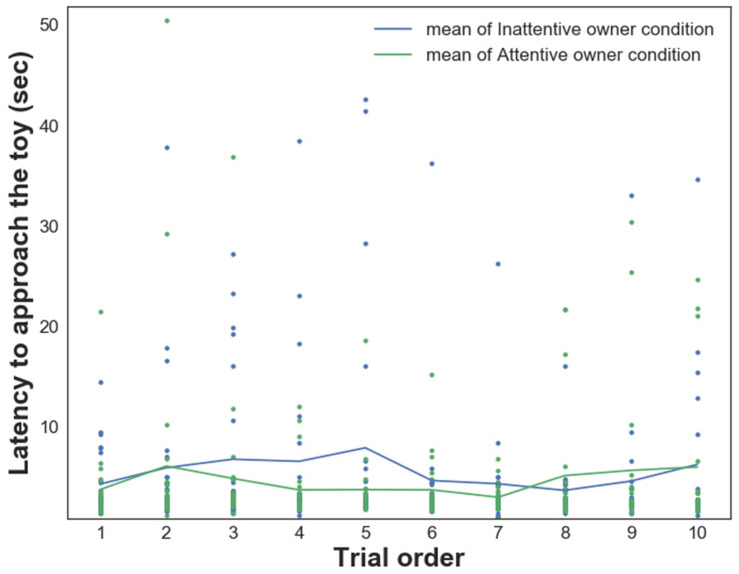
Relationship between dogs’ *Latency to approach the toy* and their owners’ visual attention in trials 1–10 in each condition.

GLMM analysis showed a significant main effect of the *Condition* on dogs’ *Latency to approach the toy* [LAT_Appr/Toy_, *F*_(__1_,_432__)_ = 6.927, *p* = 0.009; the time it took dogs to reach the toy was shorter in the *Attentive Owner* condition; Figure 2]. Moreover, the effect of the *Pet-related Avoidance Scale* was marginally significant [*F*_(__1_,_432__)_ = 3.597, *p* = 0.059]; dogs of owners with elevated PAVS tended to approach the toy object later, and there was a significant PAVS × PANXS interaction [*F*_(__1_,_432__)_ = 5.568, *p* = 0.019; dogs of owners with lower PAVS *and* PANXS tended to approach the toy object sooner]. There were no significant effects of the *Pet-related Anxiety Scale* (PANXS) and *Trial order* as well as there were no other interaction effects (*p* > 0.05 for all).

**FIGURE 3:**
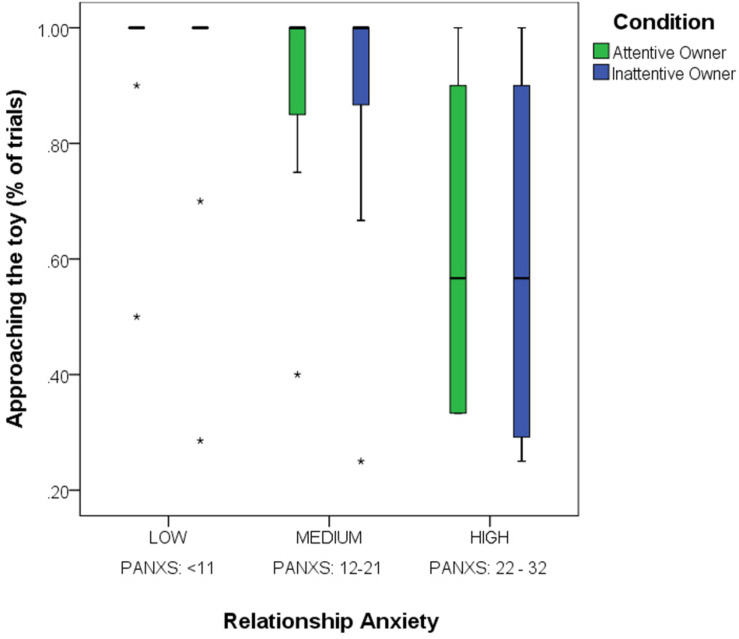
The effect of Pet-related Anxiety (PANXS) on dogs’ tendency to approach the toy object (Binary_Appr_) in the Attentive and Inattentive Owner conditions. Dogs are grouped according to their owners’ relationship anxiety (medians ± IQT and outliers).

We found a significant main effect of the *Pet-related Anxiety Scale* (PANXS) on dogs’ willingness to approach the toy [Binary_Appr_, GLMM; *F*_(__1_,_476__)_ = 4.462, *p* = 0.035; dogs of owners with higher relationship anxiety were less willing to approach the toy; Figure 3]. The main effects of the *Condition*, *Trial order*, *Pet-related Avoidance Scale* (PAVS) as well as any interaction effects were non-significant (*p* > 0.05).

Regarding the dogs’ behavior toward the experimenter, the GLMM analysis failed to show any significant main effects or interaction effects on the selected variables (relative duration of gazing toward the experimenter – WATCH_Exp_; tendency to bring back the toy to the experimenter – Binary_*Fetch/Exp*_; all *p* > 0.05).

Regarding the dogs’ behavior toward their owners, however, we found a significant main effect of the *Condition* on dogs’ tendency to involve their owners during the trial [Binary_*Fetch/Own*_, *F*_(__1_,_476__)_ = 17.747, *p* < 0.001]. Namely, dogs offered the toy object to the owner more frequently in the Attentive Owner condition (21.9% of the total trials) than in the Inattentive Owner condition (6.3% of the total trials). The main effects of the *Trial order*, *Pet-related Avoidance and Anxiety Scales* as well as the interaction effects were non-significant (*p* > 0.05).

**FIGURE 4:**
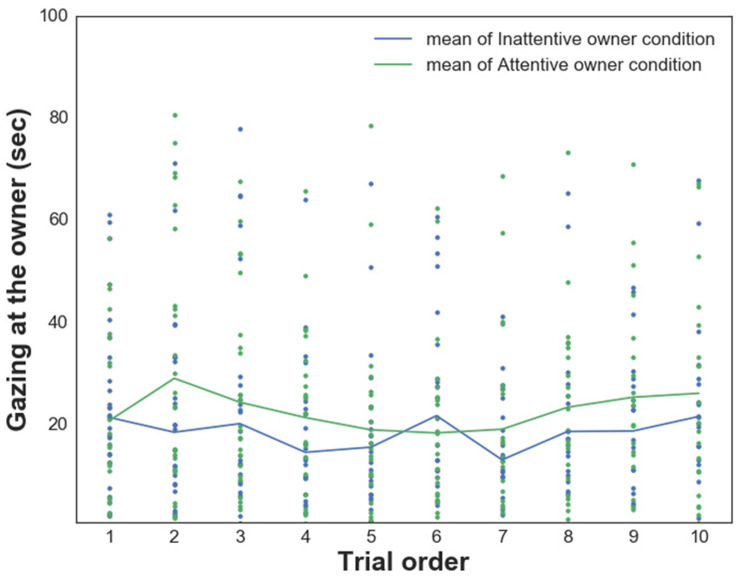
Relationship between dogs’ *Gazing at the owner* and their owners’ visual attention in trials 1–10 in each condition.

We also found a significant main effect of the *Condition* on dogs’ gazing at the owner [WATCH_*Own*_, *F*_(__1_,_476__)_ = 10.247 *p* = 0.001; dogs gazed significantly longer at their owners in the Attentive Owner condition; Figure 4]. There were no other main effects (*Trial order*, *PAVS*, *PANXS*) or interaction effects (all *p* > 0.05).

### Associations Between Sleep Physiology and Dogs’ Behavior in the Fetching Task

#### Drowsiness

**FIGURE 5:**
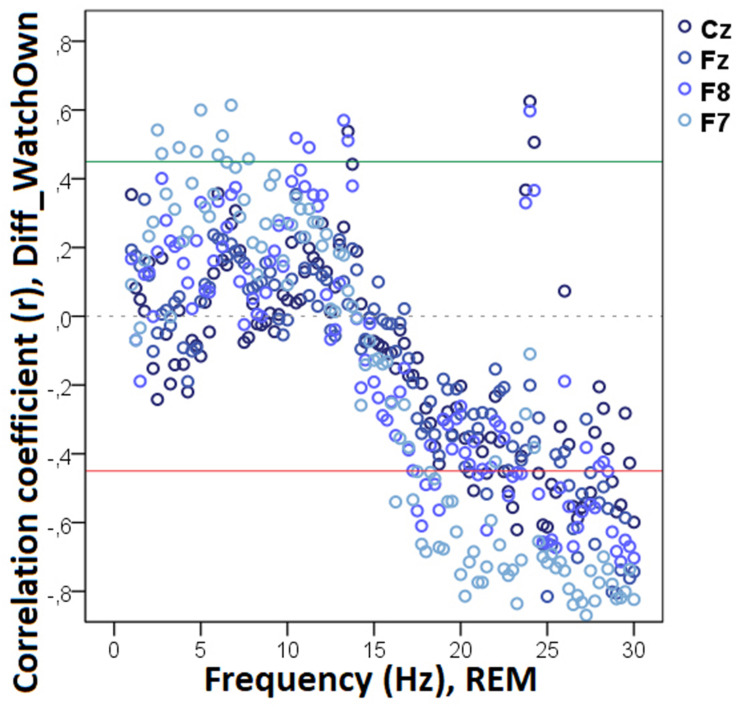
Correlation between the difference in the total number of trials during which the dog approached the toy (Attentive vs. Inattentive Owner condition) and drowsiness EEG power spectrum. Correlation coefficients for the four EEG channels (Fz, F7, F8, Cz) are shown with points above the green line (*r* = 0.45) and below the red line (*r* = -0.45) indicating significant (*p* < 0.05) associations for the given frequency bin.

**FIGURE 6:**
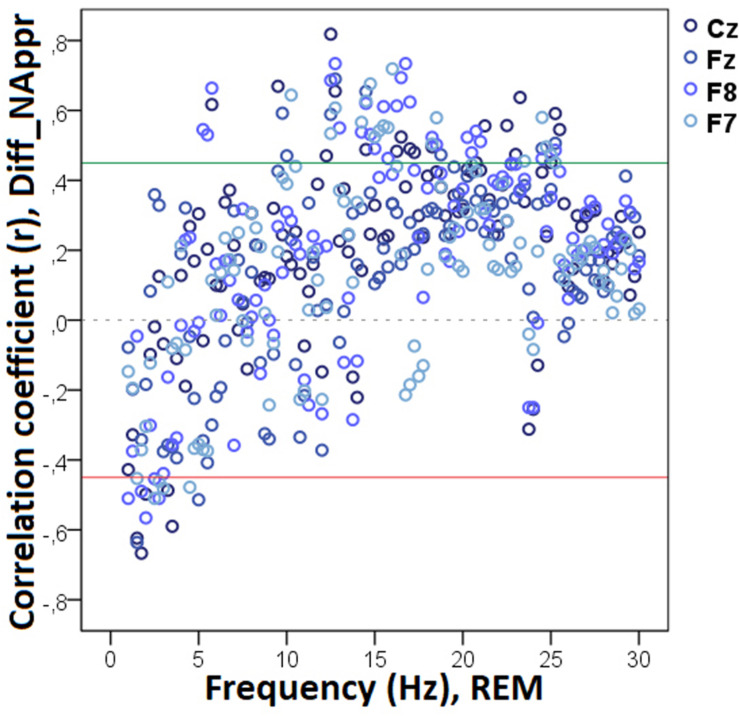
Correlation between the difference in the duration of gazing at the owner (Attentive vs. Inattentive Owner condition) and drowsiness EEG power spectrum. Correlation coefficients for the four EEG channels (Fz, F7, F8, Cz) are shown with points above the green line (*r* = 0.45) and below the red line (*r* = −0.45) indicating significant (*p* < 0.05) associations for the given frequency bin.

The bin-by-bin analysis revealed that during drowsiness there was a significant positive correlation between Diff_N_Appr_ (the difference score between the Attentive and Inattentive Owner conditions in dogs’ tendency to approach the toy) and EEG spectrum in the 20.0–20.75 Hz and 21.25–22.0 Hz (beta) ranges (Figure 5). There was also a significant positive correlation between the difference score of latency to approach the toy (Diff_LAT_Appr/Toy_) and the relative EEG spectrum power in the 8.5–9.0 Hz (alpha) range. Diff_WATCH_*Own*_ (the difference score based on the relative duration of gazing at the owner) was positively correlated with the 6.25–6.75 Hz (delta) as well as with the 11.75–12.0 (alpha) frequency ranges and showed a negative correlation with relative beta activity (in ranges: 16.75–17.75 Hz, 21.75–23.5 Hz, 24.5–30 Hz) during drowsiness (Figure 6). As regards questionnaire scores, *Pet-related Avoidance Scale* showed a negative correlation with the relative beta activity in ranges 13.75–16.0 Hz and 19.5–19.75 Hz. *Pet-related Anxiety Scale* was also negatively correlated with the EEG spectrum power in the delta range (1.5–2.5 Hz), and there was a positive relationship between this questionnaire score and the relative beta activity (14.75–15.0 Hz, 15.75–16.0 Hz, 17.5–23.5).

We found no other significant correlations of questionnaire and difference scores with delta, theta, alpha, or beta activities during drowsiness. See Table 1 for a summary of the above correlational relationships.

**TABLE 1:** Summary of the correlational relationships between the relevant EEG spectrum dimensions and selected behavioral and questionnaire measures during drowsiness.

Behavioral variable	Frequency range (Hz)	EEG channel	Direction of effect
**Drowsiness**			
Diff_N_Appr_	20.0–20.75 (beta)	F7, F8, Cz, Fz	Positive
	21.25–22.0 (beta)	F8	Positive
Diff_N_*Fetch/Own*_	–	–	–
Diff_N_*Fetch/Exp*_	–	–	–
Diff_LAT_Appr/Toy_	8.5–9.0 (alpha)	Cz	Positive
Diff_WATCH_*Own*_	6.25–6.75 (delta)	Cz, Fz, F7	Positive
	11.75–12.0 (alpha)	Fz, F7	Positive
	16.75–17.75 (beta)	F8	Negative
	21.75–23.5 (beta)	Fz, Cz, F7, F8	Negative
	24.5–30 (beta)	F8, Cz, Fz, F7	Negative
Diff _WATCH_Exp_	–	–	–
Pet-related avoidance	13.75–16.0 (beta)	F7, F8	Negative
	19.5–19.75 (beta)	F8	Negative
Pet-related anxiety	1.5–2.5 (delta)	Cz	Negative
	14.75–15.0	Cz	Positive
	15.75–16.0 (beta)	Fz, Cz, F7	Positive
	17.5–23.5 (beta)	Cz, Fz, F7, F8	Positive

#### Non-REM Sleep

**FIGURE 7:**
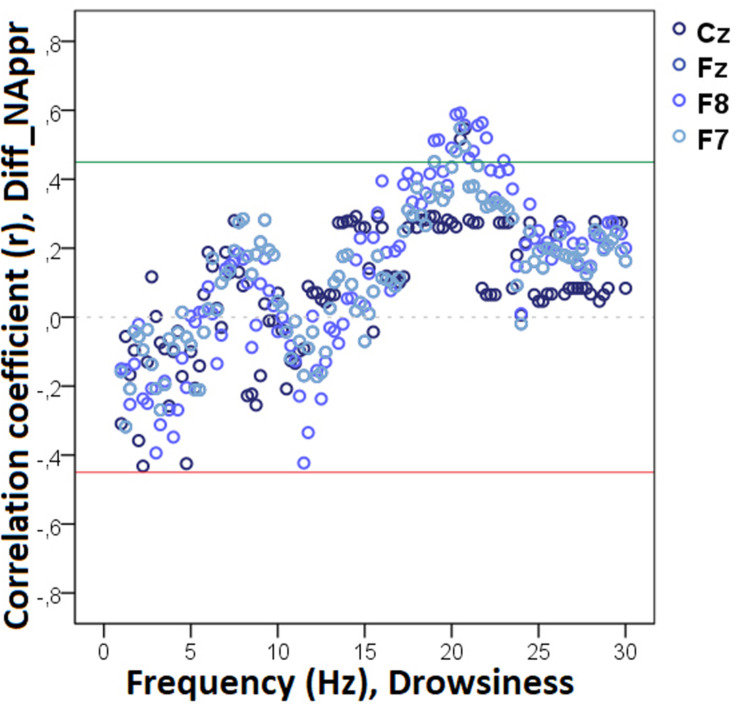
Correlation between the difference in the total number of trials during which the dog approached the toy (Attentive vs. Inattentive Owner condition) and non-REM EEG power spectrum. Correlation coefficients for the four EEG channels (Fz, F7, F8, Cz) are shown with points above the green line (*r* = 0.45) and below the red line (*r* = -0.45) indicating significant (*p* < 0.05) associations for the given frequency bin.

**FIGURE 8:**
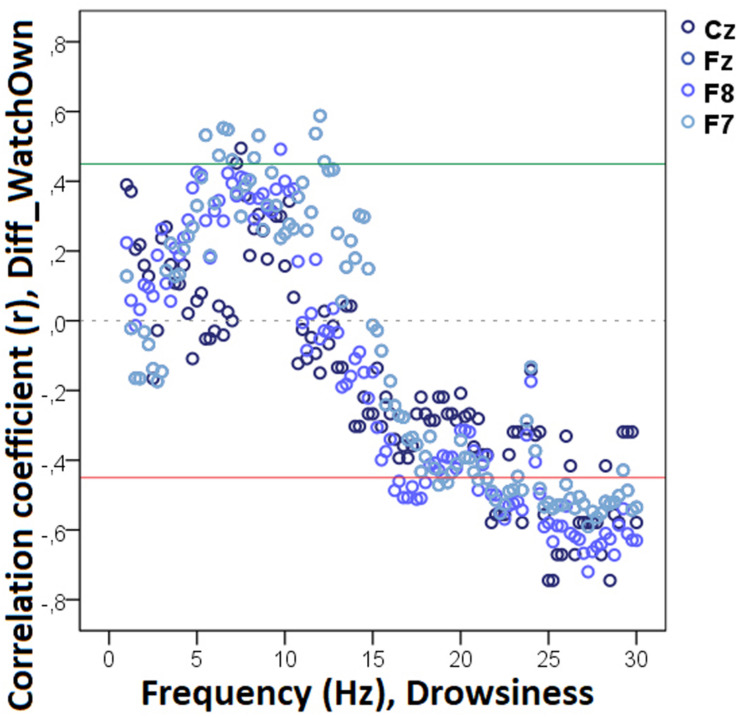
Correlation between the difference in the duration of gazing at the owner (Attentive vs. Inattentive Owner condition) and non-REM EEG power spectrum. Correlation coefficients for the four EEG channels (Fz, F7, F8, Cz) are shown with points above the green line (*r* = 0.45) and below the red line (*r* = −0.45) indicating significant (*p* < 0.05) associations for the given frequency bin.

Our analysis showed that decreased non-REM sleep delta (2.50–4.0 Hz) activity as well as increased alpha (10.0–11.0 Hz) activity were related to higher values in the difference score of the total number of trials during which the dog approached the toy (Diff_N_Appr_; Figure 7). The higher differences (AO vs. IO conditions) in the number of fully accomplished trials (Diff_N_*Fetch/Exp*_) were also related to increased non-REM sleep delta activity (2.5–2.75 Hz). Moreover, the difference score of latency to approach the toy (Diff_LAT_Appr/Toy_) was related to higher theta (6.25–7.0 Hz), alpha (8.0–8.5 Hz), and beta (11. 5–12.5 Hz) activities. There were also significant negative correlations between Diff_WATCH_*Own*_ and the relative EEG spectrum power in the 15.5–30 Hz (beta) frequency ranges (Figure 8). The analysis of the two questionnaire scores (*PAVS*, *PANXS*) indicated that increased theta (4.25–5.0 Hz) activities were related to lower scores of *Pet-related Anxiety Scale*.

We found no other significant correlations of questionnaire- and difference scores with delta, theta, alpha, or beta activities during non-REM sleep. See Table 2 for a summary of the above correlational relationships.

**TABLE 2:** Summary of the correlational relationships between the relevant EEG spectrum dimensions and selected behavioral and questionnaire measures during non-REM sleep.

Behavioral variable	Frequency range (Hz)	EEG channel	Direction of effect
**Non-REM**			
Diff_N_Appr_	2.50–4.0 (delta)	Fz	Negative
	10.0–11.0 (alpha)	Fz	Positive
Diff_N_*Fetch/Own*_	–	–	–
Diff_N_*Fetch/Exp*_	2.50–2.75 (delta)	F7	Positive
Diff_LAT_Appr/Toy_	6.25–7.0 (theta)	Fz	Positive
	8.0–8.5 (alpha)	F8	Positive
	11.5–12.50 (beta)	F7, F8	Positive
Diff_WATCH_*Own*_	15.5–30 (beta)	Fz, F7, F8, Cz	Negative
Diff_WATCH_Exp_	–	–	–
Pet-related avoidance	–	–	–
Pet-related anxiety	4.25–5.0 (theta)	F7, F8	Negative

#### REM Sleep

**FIGURE 9:**
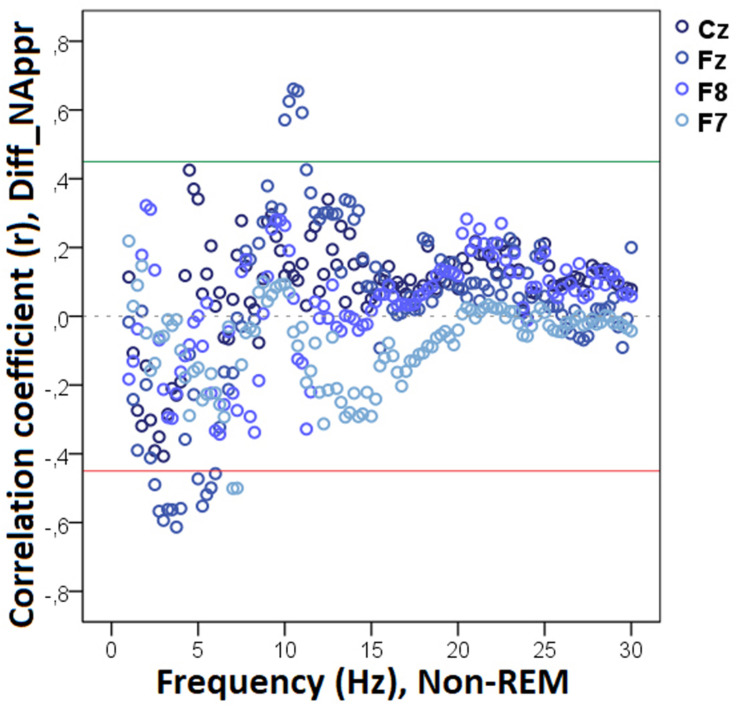
Correlation between the difference in the total number of trials during which the dog approached the toy (Attentive vs. Inattentive Owner condition) and REM EEG power spectrum. Correlation coefficients for the four EEG channels (Fz, F7, F8, Cz) are shown with points above the green line (*r* = 0.45) and below the red line (*r* = −0.45) indicating significant (*p* < 0.05) associations for the given frequency bin.

**FIGURE 10:**
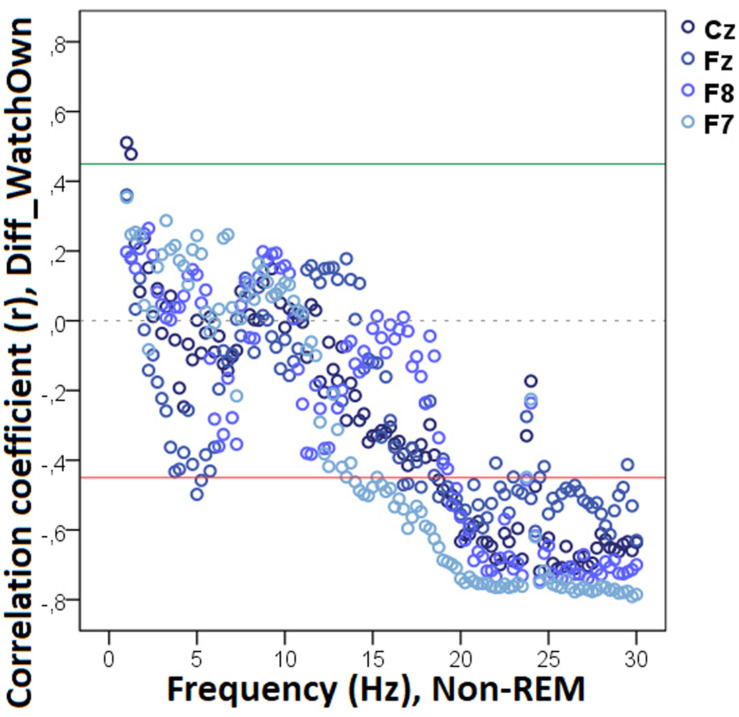
Correlation between the difference in the duration of gazing at the owner (Attentive vs. Inattentive Owner condition) and REM EEG power spectrum. Correlation coefficients for the four EEG channels (Fz, F7, F8, Cz) are shown with points above the green line (*r* = 0.45) and below the red line (*r* = −0.45) indicating significant (*p* < 0.05) associations for the given frequency bin.

Decreased REM sleep delta (1.5–1.75 Hz) activity as well as increased theta (5.25–5.75 Hz) and beta (14.25–14.75 Hz, 15.25–16.0 Hz, 16.25–17.0 Hz) activities were related to higher values in Diff_N_Appr_ (Figure 9). The difference in the number of fully accomplished trials (Diff_N_*Fetch/Exp*_) was negatively correlated with delta activity (1.5–2.0 Hz), whereas this behavioral variable was positively correlated with theta (6.25–6.75 Hz) and beta (12.75–13.0 Hz) activities. Also, in REM sleep, the higher difference score of head orientation toward the owner (Diff_WATCH_*Own*_) was related to decreased beta activity (ranges: 17.25–18.0 Hz, 18.75–21.75 Hz, 22.25–23.5 Hz, 24.5–30 Hz; Figure 10). There was a positive relationship between *Pet-related Avoidance Scale* and EEG spectrum in the 3.0–3.75 Hz (delta) frequency and a negative correlation between *PAVS* and the relative theta activity in the 6.5–6.75 Hz frequency range as well as between PAVS and relative beta activity in the 18.0–18.5 Hz range.

We found no other significant correlations of questionnaire and difference scores with delta, theta, alpha, or beta activities during REM sleep. See Table 3 for a summary of the above correlational relationships.

**TABLE 3:** Summary of the correlational relationships between the relevant EEG spectrum dimensions and selected behavioral and questionnaire measures during REM sleep.

Behavioral variable	Frequency range (Hz)	EEG channel	Direction of effect
**REM**			
Diff_N_Appr_	1.5–1.75 (delta)	Cz	Negative
	5.25–5.75 (theta)	F8, Cz	Positive
	14.25–14.75 (beta)	F8	Positive
	15.25–16.0 (beta)	F7	Positive
	16.25–17.0 (beta)	F8	Positive
Diff_N_*Fetch/Own*_	–	–	–
Diff_N_*Fetch/Exp*_	1.5–2.0 (delta)	Cz	Negative
	6.25–6.75 (theta)	Fz	Positive
	12.75–13.0 (beta)	Cz	Positive
Diff_LAT_Appr/Toy_	–	–	–
Diff_WATCH_*Own*_	17.25–18.0 (beta)	F7, F8	Negative
	18.75–21.75 (beta)	F7, F8, Fz	Negative
	22.25–23.5 (beta)	F7, Cz	Negative
	24.5–30 (beta)	Fz, F7, F8, Cz	Negative
Diff_WATCH_Exp_	–	–	–
Pet-related avoidance	3.0–3.75 (delta)	Fz, Cz	Positive
	6.5–6.75 (theta)	F7	Negative
	18.0–18.5 (beta)	F8	Negative
Pet-related anxiety	–	–	–

## Discussion

The aim of the current study was to assess the potential effect of human visual attention on dogs’ performance in a repetitive fetching task. Previous studies have shown that dogs are not only sensitive to the attentional states of humans (e.g., Virányi et al., 2004; Schwab and Huber, 2006; Kaminski et al., 2013; Brubaker et al., 2019), but have a strong propensity to follow instructions and often develop “ready-to-obey” attitudes toward humans (Topál et al., 2006, 2009; Sümegi et al., 2014). Based on these findings, we hypothesized that dogs might be susceptible to the audience effect. More specifically, we expected that dogs’ task persistence and task performance would vary according to their owners’ attentiveness.

Contrary to our expectations, we found that, independently of their owners’ attentional state, dogs were generally willing to follow the experimenter’s instructions and to comply with the fetching task. Dogs’ comparable task persistence in the Attentive and Inattentive Owner conditions is supported by the analysis of the number of trials in which they approached the toy and brought it back to the experimenter. However, a more detailed behavior analysis revealed that dogs show a somewhat human-like susceptibility to peer pressure. The effect of owners’ attention manifested itself through dogs’ toy- and owner-related behaviors. They were faster to approach the toy object in the presence of an attentive Owner, gazed significantly longer at their owners, and were more willing to offer the toy to their owners when she/he was paying attention. This finding fits previous observations that dogs are sensitive to the direction of human visual attention when they initiate interaction with humans (see, e.g., Gácsi et al., 2004). More importantly, the observed context-dependent changes in dogs’ behavior are reminiscent of effects of peer influence observed in humans (for a review, see Guerin, 1986) and generally support Zajonc’s theory of social facilitation (cf. drive theory; Zajonc, 1965). That is, we may assume that like in humans, the attentive (though passive) presence of others increases subjects’ arousal, which in turn has the potential to promote social engagement and to facilitate task-related behaviors in dogs. We should note that social facilitation (i.e., the effects of mere presence of a conspecific) has been shown in many different species including non-human primates (Ferrari et al., 2005; Dindo and de Waal, 2007; Reynaud et al., 2015), other mammals (Sherman, 1977), birds (Evans and Marler, 1994), and even fish (Karplus et al., 2006).

Another interesting thing about dogs’ behavior in the fetching task is the effect of the owner–dog relationship. Our study provides evidence that the owner’s self-assessment of his/her relationship with his/her dog may predict some aspects of dogs’ task persistence and performance. Namely, dogs of owners with higher relationship anxiety tended to approach the toy object less frequently, and dogs of owners with higher relationship avoidance and anxiety were more hesitant to approach the toy object. This finding is in line with other observations on the effects of dog–human interaction style on dogs’ task performance (Horn et al., 2012; Kis et al., 2012) and may suggest the existence of complex associations between the audience effect in dogs and characteristics of the dog–human caregiver relationship. Note, however, that this was not a direct measure of the dogs’ attachment style, and thus, it remains to be investigated which factors really determine the relationship. It seems reasonable to assume that not only relationship insecurity but also other characteristics of the pet–owner relationship as well as the dog’s personality, contribute to the behavioral effects of being watched by human caregiver. It would be interesting to examine in future studies how the dog’s attachment style is related to the pattern of behavior shown during the observation.

Note, that there were some unusual aspects of the object-retrieval task that the dogs were faced with in our study. First, the experimenter placed the target object on the floor while owners usually throw the ball in such play situations. Most dogs love to chase any thrown object because a moving object helps trigger a dog’s prey drive and, thus, raises the arousal level and contributes to the rewarding value of the game (Rooney et al., 2000). Second, everyday object-retrieval games usually require attentional engagement on the owner’s part as well as some kind of interactivity by the owner. It is reasonable to assume that, similarly to human children (Meltzoff and Decety, 2003), not only the object-directed activity (retrieval), but the interaction with the owner *per se* is socially rewarding for dogs. In our situation, however, the owner was not responsive (neither encouraged nor praised the dog) even if he/she was watching the interaction.

Analyzing the relationship between dogs’ sleep EEG spectrum and fetching task behavior is a pioneering approach to investigate the neuro-cognitive link between dogs’ personality traits and their susceptibility to the audience effect. Results show several correlations between difference scores (i.e., changes in dogs’ behavior in response to changes in owners’ visual attention) and their baseline brain activity. Thus, it appears that a dog’s individual susceptibility to the audience effect is a trait-like characteristic reflected in the EEG spectral power of both REM and non-REM sleep as well as in pre-sleep (drowsiness). The bin-by-bin analysis revealed generally consistent significant correlations across all sleep stages in case of two types of behaviors. The first one refers to the change in dogs’ task performances, that is, the differences between Attentive and Inattentive Owner conditions in dogs’ willingness to do what the experimenter commanded (to approach the toy object, *Diff_N_Appr_*). The second one, however, refers to the change in dogs’ tendency to look at their owners (*Diff_Watch_*Own*_*), which can be interpreted as changes in dogs’ propensity to initialize interaction with their owners.

Regarding the relationship between dogs’ task performances and sleep EEG spectrum, we found that the change in dogs’ tendencies to follow the experimenter’s instructions was positively correlated with the relative EEG spectrum at the higher frequencies: in the alpha range during non-REM as well as in the beta range during REM and drowsiness. Moreover, the difference score for dogs’ tendencies to approach the toy was positively correlated with REM theta, and negatively correlated with low frequency delta during REM and non-REM. The bin-by-bin analysis also revealed a consistent relative beta activity increase in correlation with the differences between Attentive and Inattentive Owner conditions in dogs’ tendency to gaze toward their owners in all sleep stages. That is, dogs characterized by higher relative beta power during sleep displayed less flexibility in adjusting their gazing behavior to their owners’ attentional state. It should also be noted that some aspects of the human–dog relationship insecurity also were reflected in the spectral characteristics of dogs’ sleep EEG. Both Pet-related Anxiety and Avoidance Scales had a robust association with the beta band, but in opposite directions: higher scores of anxiety and lower scores of avoidance scales were related to increased beta activity in drowsiness.

Based on evidence from human studies, we may assume that the increased high-frequency (mostly beta) EEG activity in dogs that tended to show a greater change in following the experimenter’s instructions and in gazing toward their owners can be interpreted as a sign of poorer sleep quality. For example higher beta power in non-REM is frequently observed in insomnia patients (Buysse et al., 2008; Marzano et al., 2008; Spiegelhalder et al., 2012), suggesting increased cortical activation resulting from nocturnal emotional and physiological hyperactivation (Spiegelhalder et al., 2011, 2012). The finding that increased alpha EEG activity in dogs is associated with reduced susceptibility to the audience effect (i.e., smaller changes in task performance between Inattentive and Attentive Owner conditions) also parallels results of human studies. That is, a reduced alpha power band is related to more efficient emotion regulation in humans (a higher resistance to immediate emotional impact of the situation; Dennis and Solomon, 2010). Moreover, theta activity during REM sleep, which positively correlated with the difference score for dogs’ tendencies to approach the toy, has been reported to be involved especially in consolidation of fear (Popa et al., 2010) and emotional (Nishida et al., 2009) memories in humans. Finally, dogs that showed a smaller response to their owners attention in their tendency to approach the toy had higher delta EEG activity, which, in a way, parallels the human finding that higher delta power in non-REM (stage 4) has been reported in antisocial patients (male subjects with borderline personality disorder; Lindberg et al., 2003).

## Conclusion

In sum, these results show that dogs accomplish the prerequisites of a human-like sensitivity to being watched, a capacity which might be linked to more complex mechanisms, such as self-presentation or reputation management, helping the two species to become effective social partners. Dogs in the present experiment, despite comparable task persistence in the Attentive and Inattentive Owner conditions, showed several behavioral differences that reflected the effect of owners’ visual attention. Their behavior was further related to trait-like parameters, such as the owner–dog relationship and dogs’ brain activity during sleep.

Susceptibility to the audience effect is one of the basic “building blocks” of social-emotional capabilities that makes human social cognition unique. It has been previously shown that functionally human-analog social behaviors emerged in dogs, including their sensitivity to the visual attention of others. Even so, any parallels to the underlying mechanisms of the audience effect in humans is still unclear. More research is needed to explore how the relationship with the owner mediates this behavior and what other factors have impact on dogs’ social sensitivity manifesting in the increased motivation to conform to the human expectations.

## Data Availability Statement

The datasets generated for this study are available on request to the corresponding author.

## Ethics Statement

The animal study was reviewed and approved by National Animal Experimentation Ethics Committee, Hungary. Written informed consent was obtained from the owners for the participation of their animals in this study.

## Author Contributions

OK, AK, and JT designed the study and interpreted the data. OK and KS prepared the study material and data acquisition. KS entered the data and prepared it for statistical analyses. OK, KS, and AK analyzed the data. JT obtained funding. OK wrote the first draft of the manuscript. JT and AK critically revised the manuscript for important intellectual content. All authors gave final approval of the manuscript version to be published and agreed to be accountable for all aspects of the work in ensuring that questions related to the accuracy or integrity of any part of the work are appropriately investigated and resolved.

## Conflict of Interest

The authors declare that the research was conducted in the absence of any commercial or financial relationships that could be construed as a potential conflict of interest.

## Footnotes

1 http://solomoncoder.com/
