# Supplementary material for: Behavioral and Neurophysiological Correlates of Dogs’ Individual Sensitivities to Being Observed by Their Owners While Performing a Repetitive Fetching Task
Source: Front Psychol. 2020 Jul 15;11:1461. doi: 10.3389/fpsyg.2020.01461 (PMC7375335; doi:10.3389/fpsyg.2020.01461)
Supplement: Supplementary file 1 [file Table_1.DOCX]

Supplementary Material

# Supplementary table

**Table S1**.

Correlation matrix of the behavioral variables. ** Significant at .01 level, * significant at 0.05 level (two tailed tests). Note that only three ‘no redundant’ variables (LAT_Appr/Toy_, WATCH_Exp_, WATCH_Own_ ) were retained for further GLMM analyses.

|  | **LAT _Appr/Toy_** | LAT _Give/Toy/Exp_ | LAT _Give/Toy/Own_ | LAT _WatchExp_ | LAT _WatchOwn_ | PROX _Exp_ | PROX _Own_ | PROX _Toy_ | **WATCH _Exp_** | **WATCH _Own_** |
| --- | --- | --- | --- | --- | --- | --- | --- | --- | --- | --- |
| **LAT_Appr/Toy_** | 1 |  |  |  |  |  |  |  |  |  |
| LAT_Give/Toy/Exp_ | ***.352^**^*** | 1 |  |  |  |  |  |  |  |  |
| LAT_Give/Toy/Own_ | *.****765^**^*** | ***.392^*^*** | 1 |  |  |  |  |  |  |  |
| LAT_WatchExp_ | .011 | ***.446^**^*** | -.083 | 1 |  |  |  |  |  |  |
| LAT_WatchOwn_ | ***.168^**^*** | ***.144^*^*** | -.029 | .017 | 1 |  |  |  |  |  |
| PROX_Exp_ | ***.251^**^*** | ***-.465^**^*** | .192 | ***-.260^**^*** | -.053 | 1 |  |  |  |  |
| PROX_Own_ | -.003 | ***.180^**^*** | ***-.303^*^*** | ***.127^**^*** | -.054 | ***-.270^**^*** | 1 |  |  |  |
| PROX_Toy_ | ***-.177^**^*** | -.024 | .080 | ***.210^**^*** | .056 | ***-.151^**^*** | ***-.374^**^*** | 1 |  |  |
| **WATCH_Exp_** | -.070 | ***-.524^**^*** | .017 | ***-.358^**^*** | -.090 | ***.420^**^*** | ***-.244^**^*** | -.023 | 1 |  |
| **WATCH_Own_** | **.146^**^** | -.078 | -.056 | .033 | ***-.142^**^*** | ***.140^**^*** | ***.539^**^*** | ***-.341^**^*** | -.042 | 1 |
